# The Extended Use of Eculizumab in Pregnancy and Complement Activation–Associated Diseases Affecting Maternal, Fetal and Neonatal Kidneys—The Future Is Now?

**DOI:** 10.3390/jcm8030407

**Published:** 2019-03-24

**Authors:** Vedran Stefanovic

**Affiliations:** Department of Obstetrics and Gynecology, Helsinki University and Helsinki University Hospital, Haartmaninkatu 2, 00290 Helsinki, Finland; vedran.stefanovic@hus.fi; Tel.: +358-50-4271230

**Keywords:** eculizumab, pregnancy, premature birth, paroxysmal nocturnal hemoglobinuria, atypical hemolytic uremic syndrome, antiphospholipid syndrome, sickle-cell anemia, HELLP, fetal kidney development, complement activation

## Abstract

Excessive complement activation is involved in the pathogenesis of many diseases and the kidney is an organ with particular susceptibility to complement-mediated injury. Apart from paroxysmal nocturnal hemoglobinuria (PNH) and atypical hemolytic uremic syndrome (aHUS), there are several other diseases with clear evidence of complement activation affecting both maternal and fetal kidneys during pregnancy and causing long-term adverse outcomes. Several novel drugs have been recently developed for blocking the complement cascade, including purified plasma proteins, new monoclonal antibodies, recombinant proteins, small molecules, and small interfering RNA agents. Eculizumab, the humanized monoclonal IgG2/4-antibody targeting C5 was approved by the United States Food and Drug Administration (FDA) and the European Medicines Agency (EMA) for treatment of two rare diseases: PNH in 2007 and aHUS in 2011. There is an increasing number of publications of successful use of eculizumab for off-label indications, e.g., in pregnant women with antiphospholipid syndrome, sickle-cell anemia, and HELLP syndrome. These severe diseases are associated with both high maternal and fetal morbidity and mortality rate and substantial prematurity. Eculizumab has considerably improved overall outcome of patients with PNH and aHUS, enabling safe pregnancy for many women. Prolongation of pregnancy and the use of eculizumab, even for only a few weeks, may protect not only maternal renal function, but also alleviate acute and long-term renal consequences of prematurity in offspring.

## 1. Introduction

Pregnancy is a unique and complex biological phenomenon with tremendous changes occurring, particularly in the maternal cardiovascular system, immune system, and kidneys [1], with the term birth of fetus with normal growth as the successful end-points. The immune system undergoes significant adaptations during pregnancy in order to ensure survival of the fetal allograft and protect mother and fetus from external threats.

An intact complement system is of essential importance for the successful maintenance of normal pregnancy and is involved in both host defence and appropriate placental and fetal development. Occasionally, excessive activation of complement may result in severe adverse pregnancy outcomes [2].

**Figure 1:**
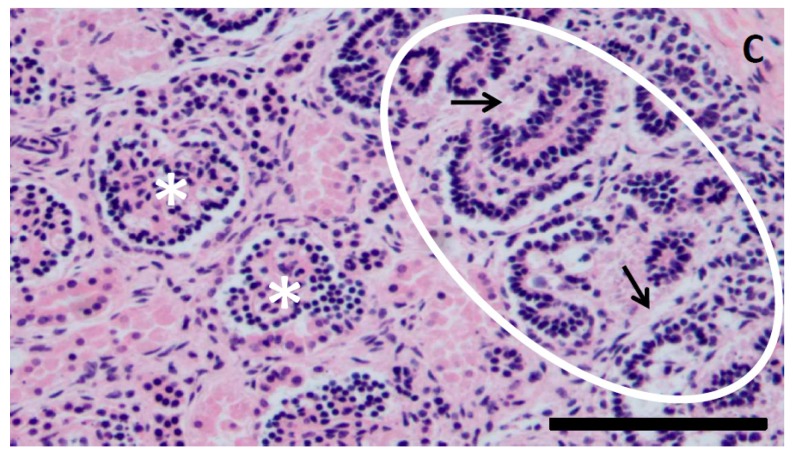
Kidney of a stillborn fetus with intrauterine growth restriction at 22 weeks of gestation. Note subcapsular (C) nephrogenic zone (encircled) with proliferating tubules (black arrows). Immature bilobed glomeruli (asterisks) lined by epithelial cells. Magnification 200×, hematoxylin-eosin staining. Scale bar 100 um. Courtesy: Dr. Jouko Lohi, Department of Pathology, Helsinki University Hospital, Finland.

Maternal kidneys have high capacity for adaptation during the pregnancy [1]. However, despite the significant improvements in obstetrical and neonatal care, burden of pregnancy, underlying maternal chronic diseases, or specific pregnancy-specific diseases may jeopardize renal function, affecting not only the mother, but also fetus and neonate, mainly due to sequelae of iatrogenic premature birth [3,4]. Kidney size and nephron number are significantly reduced in premature infants (Figure 1).

Furthermore, apart from the preterm birth, hostile intrauterine environment and aggressive neonatal treatments in extremely preterm survivors frequently cause long-term health consequences extending to adulthood including impaired neonatal renal function [5].

There are several diseases characterized by extensive complement activation, which is further triggered by the pregnancy. The kidney is an organ that is particularly susceptible to complement-mediated injury. Paroxysmal nocturnal hemoglobinuria (PNH) and atypical hemolytic-uremic syndrome (aHUS) were historically diseases in which presence pregnancy was discouraged due to the poor outcome and life-threatening complications with substantial maternal and fetal/neonatal morbidity and mortality [4,5,6,7].

Eculizumab, the humanized monoclonal IgG2/4-antibody targeting C5, was approved by the United States Food and Drug Administration (FDA) and the European Medicines Agency (EMA) for the treatment of paroxysmal nocturnal hemoglobinuria in 2007 and atypical hemolytic uremic syndrome in 2011 for treatment of these two rare diseases. Its use has significantly improved the outcome of both diseases, also in pregnant patients.

Whilst the majority of publications on eculizumab use in clinical practice involved PNH and aHUS non-pregnant patients, there is a paucity of knowledge of eculizumab use in pregnant patients, especially in diseases with off-label eculizumab use (antiphospholipid syndrome, sickle-cell anemia, and preeclampsia/HELLP syndrome) (Table 1). Additionally, most of the publications have been focused on maternal, but not on fetal and neonatal outcome.

Prolongation of pregnancy, even for only a few weeks could improve not only maternal renal function, but also alleviate renal consequences of prematurity in offspring.

The purpose of this narrative review is to update relevant recent literature on eculizumab use in “classical” diseases related to the complement activation (PNH and aHUS) in non-pregnant patients, and to focus on its use in pregnant women affected PNH and aHUS with special emphasis on the adverse renal outcomes in mothers and their offspring.

We will particularly focus on renal consequences of the premature birth that affect a substantial proportion of women not only with PNH and aHUS, but also antiphospholipid syndrome, sickle-cell anemia, and preeclampsia/HELLP syndrome.

## 2. Antiphospholipid Syndrome

Antiphospholipid syndrome (APS) is a clinical condition characterized by recurrent thrombosis and pregnancy morbidity in the presence of antiphospholipid antibodies (aPL). It is well-acknowledged that pregnant women with APS are an extremely high-risk group for adverse outcome affecting mother, fetus, and a newborn, including early miscarriage, stillbirth, fetal growth restriction, neonatal asphyxia, and prematurity with consequent serious perinatal mortality and morbidity. The study of Jeremic et al. [18] that included 55 pregnant women with APS reported pregnancy loss and prematurity rates of 18.2% and 31.8%. Also, significantly more patients with APS experienced thrombocytopenia, pregnancy losses, intrauterine growth restriction, preeclampsia, and perinatal asphyxia compared with the control group that included 55 heathy pregnant women. Similar results were obtained in other cohorts of different sizes [19,20,21,22].

At present, therapy of pregnant women with APS is based on preventing thrombosis by acetylsalicylic acid (ASA) and/or low molecular weight heparin (LMWH), but anticoagulation is only partially successful in decreasing adverse pregnancy outcomes.

The PREGNANTS study [19] reported an increased risk of obstetric complications and lower live birth rate when more than one antiphospholipid antibody is present in women with primary antiphospholipid syndrome. Additionally, the study showed that despite therapy with low-dose ASA and prophylacticLMWH, the chance of a liveborn neonate was only 30% for triple-positive women.

There is an emerging experimental and clinical evidence supporting the role of complement cascade activation in the underlying pathophysiology of aPL-induced pregnancy loss and thrombosis. Girardi et al. hypothesized that complement activation is a central mechanism of pregnancy loss in APS [8]. They tested this in pregnant mice that received human IgG containing aPL antibodies. Complement component C5 (and particularly its cleavage product C5a) and neutrophils were reported to be key mediators of fetal injury. It was demonstrated that antibodies or peptides that block C5a-C5a receptor interactions prevent pregnancy complications. Based on their results, authors conclude that identifying complement-related markers that predict high risk for fetal loss would enable us to translate knowledge about the mechanisms of complement-mediated disease into complement-targeted therapeutics that may prevent, arrest, or modify the deleterious effects of aPL antibodies.

Romay-Penabad et al. [9] examined the effects of rEV576 (coversin), a recombinant protein inhibitor of complement factor 5 activation, on APL antibody-mediated tissue factor up-regulation and thrombosis in non-pregnant mice by administering either IgG from a patient with antiphospholipid syndrome (APS) or control IgG from normal human serum. Animals treated with IgG-APS had significantly higher titers of anticardiolipin antibodies and anti-β2GPI and showed significantly larger thrombi compared with other groups. On the contrary, mice treated with IgG-APS/rEV576 had significantly smaller thrombi than those treated with IgG-APS/phosphate buffer. The results of this research confirmed involvement of complement activation in antiphospholipid antibody-mediated thrombogenesis and suggested that this effect might be ameliorated by complement inhibition.

There are only two case reports on eculizumab use in pregnant women with APS. Gustavsen et al. [10] reported in details a case of 22-year- old primigravida with triple positive aPL antibodies and a history of arterial thrombosis treated with by-pass grafting and digital amputations. Additionally, recurrent episodes of thrombosis were treated with percutaneous transluminal angioplasty, and an episode of microemboli resolved with intensified anticoagulant treatment that continued during the pregnancy. She was admitted in the third trimester with painful ulcerations of ischemic origin in her right leg. Due to multiple previous arterial thromboses and ongoing ischemia during pregnancy, it was estimated that the risk of developing catastrophic APS in relation to pregnancy, delivery, and puerperium was significant. Eculizumab was administered twice: 600 mg 8 days before delivery (day 0) in addition to prophylactic antibiotics and thereafter a 2nd dose of 600 mg was infused on day 7. The following day (day 8), at 32^+4^ gestational weeks, cesarean section was performed with the birth of a healthy child.

In this case, complement activity surprisingly increased to normal levels within a week after both doses of eculizumab despite the evidence of complete inhibition of complement activity after each infusion. Authors speculate that pregnancy-induced changes might have influenced the pharmacodynamics and pharmacokinetics of eculizumab. The lesson learnt from this report is that an individual approach to the complement inhibition and adequate monitoring of the treatment effects should be implemented to enable optimal dosage and duration of eculizumab treatment in such patients.

Rovere-Querini et al. [11] described a pregnant woman who had previous thrombotic events, a history of abortions, and triple persistent positivity for aPL and abruptly developed microangiopathic hemolytic anemia, renal insufficiency, and thrombocytopenia at 30^+6^ weeks of pregnancy despite previous treatment with rituximab and current treatment with hydroxycholoquine, therapeutic dosage of LMWH along with low dose ASA. The onset of severe clinical situation was precipitated by acute hemorrhage from a vulvar angiokeratoma. In less than a week, hemolysis, thrombocytopenia, and renal functions worsened despite continuous infusion of antithrombin III concentrate. The patient had clear laboratory evidence of C3 activation. Eculizumab was administered and pregnancy was safely continued for 9 days after which cesarean section was performed due to the fall of platelet count. A healthy baby with favourable outcome was born. The second eculizumab infusion was administered a week after the first treatment with rapid normalization of platelet count, renal function, and hemoglobin level.

Authors concluded that eculizumab represented a worthy addition to the existing treatment regimen, which may prevent the rapid deterioration of clinical conditions and enable safe pregnancy prolongation for reasonable and clinically relevant period as well as enable safe and rapid recovery from pregnancy.

Women with APS receiving intense anticoagulant therapy are at increased risk for peri- and postpartum hemorrhage. On the other hand, cesarean section and cessation of anticoagulant treatment would considerably increase the risk of triggering catastrophic APS. Introduction of eculizumab in these cases seems biologically justified and plausible.

## 3. Sickle-Cell Trait/Anemia/Disease

Sickle cell disease (SCD) is a serious and painful disease with the consequent significant morbidity and mortality. Also, pregnancy in a patient with SCD is associated with high maternal and fetal morbidity and mortality; the maternal and fetal death rates have been reported to be as high as 20% [23,24].

Renal involvement in patients with asymptomatic and manifest SCD is frequent with proteinuria as a common feature of sickle-cell nephropathy (SCN), which may progress to renal insufficiency and end stage renal disease [25]. A systematic review and meta-analysis [26] which included the pooled sample size of more than 26,000 pregnancies in women with SCD showed a strong association between SCD and adverse outcome for both mother (including maternal mortality and pre-eclampsia) and fetus/newborn (perinatal death, preterm delivery, and small for gestational age infants). The recent extensive review of the current literature regarding pregnancy outcome of women with sickle-cell anemia showed high maternal mortality, particularly in the intra-and post-partum periods [27]. It has been demonstrated that both maternal and fetal mortality may be reduced by improvements in transfusion policy.

Knowledge of these risks has contributed to the implementation of a multidisciplinary management of such pregnancies including the early detection and treatment of pregnancy and postpartum complications, follow-up, appropriate pain management protocols, and individual blood transfusion programs to each pregnant patient. However, no prospective controlled trials have been conducted.

An important component of hemolysis in SCD mechanism may also be complement activation, which is a rather neglected entity in SCD. The first reports of complement activation in patients with asymptomatic [28,29] and symptomatic patients [30] with sickle-cell anemia have been published almost four decades ago. Most of the publications has been investigating C3 complement component. After the relative paucity of investigations regarding complement activation role in SCD, the recent publications rebooted this issue.

Gavriilaki et al. [12] studied for the first time novel markers of SCD that have been shown to reliably detect complement activation in the serum of patients with thrombotic microangiopathies [12]. Increased complement activation was demonstrated in a portion of patients, especially older patients and those with higher HbS levels. They evaluated in vitro the efficacy of complement inhibition by eculizumab in the modified Ham test and demonstrated that mixing eculizumab-containing serum with complement-activated sera abolished complement-mediated cell killing in a dose-dependent relationship that was consistent across the 3 patients tested. Also, soluble C5b-9 levels were significantly increased compared to normal human serum suggesting that complement dysregulation might be an additional factor in the complex pathophysiology of SCD in steady state. Similarly, Chapin et al. reported increased soluble C5b-9 levels in patients with SCD [31].

Chonat et al. reported atypical hemolytic uremic syndrome in a patient with sickle-cell disease with respiratory and renal insufficiency unresponsive to plasmapheresis successfully treated with eculizumab [13]. The patient showed clear evidence of complement activation demonstrated by increased levels of sC5b-9 levels retrospectively analyzed from stored plasma prior to plasmapheresis. After eculizumab treatment and improvement of clinical symptoms and laboratory baseline, sC5b-9 levels normalized. Additionally, sequence analysis of the complement regulatory genes revealed a heterozygous mutation of CFB (c.724A>C, p.I242L), previously reported to be associated with aHUS [32].

This case demonstrated that patients with SCD may develop complement-mediated thrombotic microangiopathy i.e., aHUS, particularly when an underlying genetic defect in complement regulation is present. The obvious benefit of eculizumab clearly emphasized the pathogenicity of complement activation in this rare disease.

The only report of use of eculizumab for the management of hyperhemolysis in pregnancy in sickle cell disease was published by Kirui et al. [14]. This case report described a pregnant woman with HbSS who presented with hyperhemolysis at 25 gestational weeks, seven days after a red cell transfusion for a vaso-occlusive crisis. Her anemia was worsening despite methylprednisolone and immunoglobulin treatment. Eculizumab treatment resulted in resolution of the hemolysis, with safe delivery at 34 weeks gestation. Authors suggest that eculizumab should be accessible and considered in severe cases of hyperhaemolysis which are refractory to standard treatment in pregnant women with SCD refractory to conventional therapy.

A systematic review of clinical outcomes associated with sickle cell trait (SCT) revealed a positive association between SCT and chronic kidney disease among other adverse health outcomes, such as pulmonary embolism and other complications [33].

Despite of some improvement in the management, SCD remains a serious disease with high mortality rate [34]. Early recognition of patients with increased complement activation may facilitate prompt intervention. Prospective studies are needed to better understand the role of complement in the pathophysiology and therapy of SCD.

## 4. HELLP Syndrome

HELLP syndrome (Hemolysis, ELevated liver enzymes, and Low Platelets) is usually considered as a severe variant or continuum of pre-eclampsia and is observed in 0.8% of all pregnancies. HELLP complicates 10% to 20% of pregnancies with pre-eclampsia but may also occur without it.

Although the exact pathogenesis of HELLP still remains obscure, it is usually believed that abnormal placentation, endothelial dysfunction, and release of vasoactive substances in the first trimester are the main etiological factors [35]. Interestingly, alternative pathway of complement activation coincides with the critical weeks of placentation [36].

Qing et al. showed that targeted inhibition of complement activation prevents features of preeclampsia in mice by prevention of oxidative stress and placental dysfunction [37].

A small cohort of 16 women with HELLP syndrome treated at academic tertiary care demonstrated complement activation in 9 laboratory parameters [15]. Interestingly, in the majority of cases, classical pathway of complement activation identified by a decrease in C4 levels was predominant. Furthermore, one of the pregnant women was diagnosed with APS and 5 patients had a partial expression deficiency of CD 55, which regulates and block the complement membrane attack complex. Of note, CD 55 expression deficiency is commonly used as a marker of PNH.

The study of Ma et al. showed increased C5a deposition in macrophages and C5a receptor (C5aR) expression in trophoblasts of pre-eclamptic placentas compared to placentas from normal pregnancies [38]. They also demonstrated that maternal C5a serum level was increased in women with pre-eclampsia and was positively correlated with maternal blood pressure and arterial stiffness. Authors conclude that the placental C5a/C5aR pathway contributed to the development of pre-eclampsia by regulating placental trophoblast dysfunction. Hypothesising that HELLP syndrome is a 2-hit disease similar to, requiring both genetic susceptibility [39,40] and an environmental risk factor, Vaught et al. demonstrated with the modified Ham assay that HELLP syndrome is associated with increased alternative component of complement activation [16]. This study also reported that mixing HELLP serum with eculizumab-containing serum resulted in a significant decrease in cell killing compared with HELLP serum alone. Authors thus suggest the modified Ham test as a promising tool to identify patients with increased complement activation who might benefit from complement activation blockade. Data of this study demonstrated striking similarities between HELLP and aHUS.

There are several retrospective cohort studies reporting perinatal and neonatal outcome in pregnancies with HELLP. Gul et al. reported in the cohort of 367 consecutive severe pre-eclampsia (29% of which had HELLP syndrome) that perinatal mortality and neonatal morbidity and mortality were similar in HELLP syndrome compared with severe preeclampsia-eclampsia without HELLP [41]. However, overall fetal mortality was higher in HELLP syndrome with no regular prenatal care. In HELLP group there were 10.3% stillbirths and perinatal mortality was 16.8%.

A retrospective cohort study of Roelofsen et al. investigated maternal-fetal outcome of infants born after pregnancies complicated by (H)ELLP syndrome [42]. Total perinatal mortality was 17.6%. After 18 months, four infants had major handicaps, making a total adverse outcome of 22.8%. This study demonstrated that early gestational age influenced outcome of the infants most.

Another cohort of 126 cases pregnancies with HELLP was reported by Erdemoğlu et al. showed that the stillbirth rate was 14.2% and 11.9% of infants died in the early neonatal period [43]. Of note, acute renal insufficiency complicated early neonatal period in 11.1% and a very high maternal mortality rate was reported (8%).

The retrospective study from Thailand on cohort of 213 preeclamptic women (7.5% with HELLP) demonstrated the stillbirth rate of 1.4% in severe preeclampsia and HELLP [44]. Intrapartum death rate was also considerably high in the HELLP group (6.4%) but with no statistical significance compared to women with mild preeclampsia.

Kim et al. investigated neonatal outcome after preterm delivery with HELLP and compared it to those of pregnancies with preterm preeclampsia and normotensive preterm pregnancies [45]. They reported 19.5% of neonatal deaths compared to 2% in the group of normotensive subjects. There were none stillbirth in the normotensive group, but 4.8% rate of stillbirth was reported in the HELLP group.

Hungarian cohort of 50 pregnancies with HELLP showed the incidence of premature birth of 40.7% and 7.4% neonatal mortality rate [46]. Additionally, this study showed that pregnancies with history of previous HELLP syndrome carry substantial risk for HELLP syndrome recurrence and also the development of chronic hypertension.

Furthermore, there is an increased subsequent risk of chronic kidney disease (CKD) associated with hypertensive disorders. Women with pre-eclampsia were also found to have CKD earlier than normotensive women [47].

Although the expectant care in pregnancies with HELLP may gain few days and slightly improve the neonatal outcome, it is associated with severe maternal complications. In most of the cases, prompt delivery is indicated [48]. The HELLP syndrome is associated with pregnancy-related acute kidney injury (Pr-AKI) in 7% to 36% of cases, and is often mistakenly classified as a preeclampsia/eclampsia continuum, even though 20% of cases do not have antecedent hypertension or proteinuria as essential criteria for these pregnancy disorders [3,35]. Although Pr-AKI shows decreasing incidence in developing countries, recent reports showed increased incidence in Canada and USA [49,50]. The reason may be not only increasing sensitivity of AKI diagnosis with close obstetric care, especially in high risk pregnancy, but may also be attributed to factors such as older maternal age, increased proportion of pregnancies with pregnancies with hypertensive disorders, and women who decide to conceive despite of underlying chronic kidney disease.

HELLP syndrome is a leading cause of P-AKI that occurs in up to 15% of cases of HELLP syndrome. AKI associated with HELLP syndrome, even in its severe forms requiring dialysis, is usually transient However, 10% of patients, especially those with pre-existing hypertensive or renal disease, progress to CKD [35,51,52]. HELLP syndrome shares complement gene mutations with aHUS and both conditions are characterized by elevated C5a and C5b-9 levels [53].

There is only one well-documented case report on eculizumab use in pregnancy with HELLP, although it has not been officially approved for this indication [17]. Prolongation of pregnancy from 26^+3^ to 28^+6^ (17 days) was achieved. In this case, reduction of soluble C5b-9 in plasma and urine correlated with clinical improvement, and resolution of hemolysis, thrombocytopenia and liver inflammation [54,55] along with a prolonged pregnancy. A conference abstract from the same institution [56] as the previous case report included also another patient with preeclampsia associated HELLP, whose clinical symptoms and laboratory parameters showed dramatic improvement after single eculizumab dose. The pregnancy was prolonged for three days. Cesarean section was done due to stable thrombocytopenia. This report is lacking details, so only one well-documented case in the literature remains.

There is a considerable improvement in neonatal outcome for each week of gestation in this group of patients, especially when timing of antenatal steroids and magnesium sulphate neuroprotection is optimal [57,58]. It may be possible that in the near future, eculizumab will be a drug of choice for selected pregnant women with the clear signs of complement activation in severe pre-eclampsia/HELLP. Its use may be particularly helpful among women with mutations in complement regulatory proteins, which are found in 8–18% of women with severe preeclampsia [59].

## 5. Paroxysmal Nocturnal Hemoglobinuria (PNH)

Paroxysmal nocturnal hemoglobinuria is a rare acquired stem-cell clonal disease that results from uncontrolled complement system activation characterized mostly by the intravascular hemolysis. Paradoxically, PNH name is actually incorrect; hemolysis is constant, not paroxysmal, it does not occur only during night and most of the patients do not have hemoglobinuria. A somatic mutation of the *PIG-A* gene in the hematopoietic stem cells leads to a deficiency in the glycosylphosphatydilinositol (GPI) membrane-anchoring proteins CD55 and CD59 that are complement inhibitors and makes PNH erythrocytes susceptible to the complement-mediated lysis [60]. As a result of free hemoglobin generation and depletion of nitric oxide, vasoconstriction and platelet activation occur [61]. The precise incidence of PNH has not been well-documented, but it is estimated to be 1.5 to 2 cases per million of the population per year [62]. The incidence during pregnancy is not known. PNH represent serious prothrombotic condition with severe renal insufficiency and life-threatening multiple organ failure. Patients with PNH have six-fold greater risk of chronic kidney disease compared to the general population, which is contributing to the premature mortality and contributes to up to 18% of PNH-related deaths [61,62,63]. Until recently, the treatment of PNH was mainly supportive including blood transfusions, anabolic steroids and corticosteroids along with the thrombosis prophylaxis. An allogenic hematopoietic transplant reserved for selected patients [64].

The development and approval of eculizumab that inhibits terminal complement activation changed the treatment landscape dramatically with the improvement of quality of life and overall survival. One of the largest single institution cohorts of patients with PNH was described in Spanish study with 56 patients recruited within 40 years, 16 of which have been treated with eculizumab [65]. The median survival rate of the patients from the beginning of the follow-up was only 11 years. Of 16 patients treated with eculizumab none has presented clinically relevant infection. There were not eculizumab-treated pregnant women with PNH in this cohort. The prevalence of renal failure in the whole cohort was high varying from 14% in subclinical form of disease to 44.8% in those with classic PNH. Eculizumab appeared as a safe drug in this case series of 16 patients, provided improvement in the patients’ quality of life and the disappearance of clinical symptoms. The efficacy and safety of the eculizumab treatment in PNH was demonstrated in the double-blind randomized controlled trial (TRIUMPH) [66] and open-label, non–placebo-controlled SHEPHERD study on the broader PNH population [67]. Of special interest, eculizumab particularly improved a renal function in patients with PNH, especially if administered early in the course of disease before the kidney is more severely impaired. The same results were demonstrated in a retrospective analysis from the Spanish PNH registry including 128 patients [68]. Authors concluded that treatment with eculizumab in PNH has a beneficial effect on renal function, preventing acute renal failure and progression to chronic renal failure [68].

Apart from the dramatical improvement in clinical symptoms of the patients with PNH, the treatment with eculizumab also improved nearly all aspects of the quality of life. Long-term safety and efficacy data on eculizumab have been reported by two studies [63,69]. Both studies yielded similar results and generally demonstrated that eculizumab was well tolerated, with no evidence of cumulative toxicity and a decreasing occurrence of adverse events over time. Al-Ani et al. reviewed some special considerations pertaining to the use of eculizumab in PNH [70].

From the historical perspective, pregnancy has been discouraged in patients with PNH due to the extremely high rate of adverse outcomes including maternal mortality in up to 20% of cases with thromboembolism as the major cause of death [6].

Of importance, fetal and neonatal mortality were reported to be as high as 9%, mainly due to the high rate of extreme prematurity with only half of the pregnancies progressing to the term [6]. Intravascular hemolysis as the key clinical feature of PNH is frequently more severe during the pregnancy compared to the non-pregnant state and pregnancy itself as a highly thrombogenic state complicates further the features of this disease.

Data on the use of eculizumab in women during pregnancy are rare. Apart from a dozen of case reports on the use of eculizumab in pregnant women with PNH, the most comprehensive study of efficacy and safety of eculizumab in pregnant patients with PNH was carried out by Kelly et al. [71]. This study examined 75 pregnancies in 61 women identified from the International PNH registry. Birth and developmental records of children were also carried out by the questionnaire. There were no maternal deaths and three fetal deaths occurred (4%). Requirements for blood transfusions increased during pregnancy. In 54% of pregnancies, the dose or the frequency of eculizumab treatment had to be increased, which was expected due to the concomitant physiological increase in complement activation during the second and especially third trimester. There were no thrombotic events during pregnancy; two of the thrombotic events were recorded in the postpartum period.

The prematurity rate was 29% which was rather high, but much lower than in the historical cohorts before the eculizumab era. Neonatal complications were mostly due to the prematurity. There were no cases of severe neonatal infections. This may be explained partly by the results of our study which demonstrated that eculizumab treatment does not affect the complement system activity of the newborn [72]. Eculizumab was not detected in any of the 10 breast-milk samples. Developmental assessment of children was performed at median age of 31 months in 64 children. Except one child with slightly delayed speech development, no particular neurodevelopmental problems were observed. However, proper comparison of pregnancies with PNH treated with eculizumab with those before eculizumab use is almost difficult due to the potential selection bias and retrospective nature of the studies published. It is unlikely that randomized trials will be ever conducted due to ethical reasons.

## 6. Atypical Hemolytic Uremic Syndrome (aHUS)

Atypical hemolytic uremic syndrome (aHUS) is a rare and life-threatening disorder mostly caused by inherited defects of the alternative pathway of complement characterized by microangiopathic hemolytic anemia, thrombocytopenia and acute kidney injury. It may occasionally be triggered by pregnancy (p-aHUS) and is complicating 1:25,000 pregnancies, particularly in the postpartum period [73,74]. Most of the studies on aHUS included non-pregnant patients, while studies describing p-aHUS are rare and mostly describe maternal outcomes with little focus on the neonatal outcome. While Fakhouri et al. reported that the risk for pregnancy-associated aHUS was highest during a second pregnancy [75], two other studies demonstrated that pregnancy-associated aHUS occured in majority of cases during the first pregnancy [73,76]. The latter retrospective international study that included 87 women with p-aHUS revealed the same frequency of complement gene variants and similar severity at onset and during follow-up as those with aHUS not related to pregnancy. This study reported that 62% reached end-stage renal disease by 1 month and 76% by last follow-up. None of the patients received eculizumab. A study of Fakhouri et al. reported no differences in outcomes, comparing patients with pregnancy- related and non- pregnancy-related aHUS [75]. The Vienna Thrombotic Microangiopathy Cohort retrospectively and prospectively investigated maternal and fetal/neonatal outcomes in 27 pregnancies of 14 women with aHUS [77]. While majority of pregnancies resulted in a term live birth (70%), two preterm live births, two preterm stillbirths and four early spontaneous abortions before 21 weeks of gestation occurred. Although short-term renal outcome was good in majority of women, long-term renal outcome was poor with 4/14 women having CKD stage 1-4, 5/14 had received a renal allograft, and 3/14 were dialysis-dependent at the end of this study.

A systematic review of eculizumab for atypical aHUS demonstrated its effectiveness and long-term safety in the treatment of this disorder [78]. Coffiell et al. evaluated longitudinally the effect of terminal complement blockade in patients with aHUS with eculizumab by measuring biomarkers of cellular processes and compared them with those of healthy volunteers [79]. In patients with aHUS, both with and without identified complement mutations, markers were significantly elevated at baseline. During ongoing eculizumab therapy, a significant reduction of marker levels in both subsets of patients was observed. The aforementioned retrospective study from Spain analyzed clinical and prognostic data of 22 p-aHUS patients under different treatment [73]. Seventeen patients had plasma treatments with positive renal response in only 3 cases, while 10 patients were treated with eculizumab with an excellent response.

Historically, management of pregnancy in aHUS patients has been challenging and pregnancy was discouraged. However, women with aHUS who are receiving eculizumab at present have an improved quality of life and are more likely to consider pregnancy.

Reports on the use of eculizumab during pregnancy are mostly single case reports [80,81,82]. The results of those studies are displayed in a recently published narrative overview of eculizumab use in pregnancy by Sarno et al. [83]. There was a huge difference in time of commencing eculizumab treatment, doses, infusion intervals and duration of the therapy. The largest study described six pregnancies with ongoing eculizumab treatment in three pregnant women [84]. All women had reduced glomerular function at the beginning of their pregnancies. Although eculizumab treatment prevented relapse of aHUS in all pregnancies, it did not prevent HELLP syndrome in one patient and pre-eclampsia in two other patients.

## 7. Preterm Birth and Its Influence on the Renal Development and Function in Offspring

Preterm birth (PTB), defined as birth before 37 gestational weeks is the leading cause of neonatal morbidity and mortality [5]. Furthermore, extremely PTB is defined as occurring at less than 28 weeks, very preterm delivery occurring between 28 and 32 weeks, and moderate to late PTB occurring from 32 through completed 36 weeks. These definitions have important clinical relevance as morbidity and mortality dramatically increase as the duration of gestation decreases. Also, obstetric management differs with gestational age.

According to the mode of onset of delivery, preterm births can be classified as iatrogenic or spontaneous. Iatrogenic preterm birth is a result of induced labor. Maternal and fetal pregnancy complications, among them preeclampsia and HELLP as well as rare diseases (PNH, aHUS, APS, and SCD) are among the reasons for rising prevalence of iatrogenic preterm delivery. Furthermore, due to the improved neonatal care and survival of extremely preterm infants, the ratio of iatrogenic and spontaneous preterm birth have been changed during the recent decade [85]. Infants born following medically indicated preterm birth are at a two-fold higher risk of neonatal mortality as compared with infants born following spontaneous preterm birth [86].

Stevens et al. examined economic burden of preeclampsia in the United States [87] and demonstrated that in 2012, the cost of preeclampsia within the first 12 months of delivery was $2.18 billion ($1.03 billion for mothers and $1.15 billion for infants), and was disproportionately borne by births of low gestational age. Authors demonstrated that a reduction in gestational age by 2 weeks increased costs for all infants by $1.15 billion. Conversely, mean decrease in costs per infant for each additional week of gestation was the steepest from 23 to 26 weeks, demonstrated by Manuck et al. [88]. After 25 weeks, the length of stay decreased significantly with each additional completed week of pregnancy. Among babies delivered from 26 to 32 weeks of gestation, each additional week in utero reduced the subsequent length of neonatal hospitalization by a minimum of 8 days. Neonatal mortality was 44% among infants delivered at 23 gestational weeks compared to 12%, 8%, and 2% among those born at 25, 27, and 28 gestational weeks, respectively. Major morbidity among survivors decreased from 52% at 26 gestational weeks to 23% at 28 gestational weeks.

These data provided valuable information regarding a spectrum of neonatal outcomes for each week of completed pregnancy. Considering the cost of eculizumab treatment for aHUS and HELLP, prolongation of such pregnancies for a few weeks might be also cost-effective.

The population-based study from Finland evaluated the effect of gestational age and prematurity-related morbidities on hospital costs and cost per quality-adjusted life-year (QALY) during the first 4 years of life [89]. Authors demonstrated that the prematurity-related morbidities were associated with increased costs in the very preterm population and also that initial hospital stay accounted for most of the costs. Additionally, prematurity-related morbidities were the most expensive after the initial hospitalization.

A common feature of extremely premature birth is disruption of organogenesis and arresting in branching organs. The development of fetal kidneys shares similar patterns with organogenesis of the lungs, pancreas and vascular tree [90].

Nephrogenesis in the human fetus begins during the sixth week of pregnancy and continues through the 36th week with the majority of nephron development occurring during the last pregnancy trimester. Studies in mice have demonstrated that premature birth decreases nephron number and decreases kidney volume compared to mice born at full gestation with the consequent developing of CKD with hypertension and albuminuria [91]. Similar results have been obtained in experiments on nonhuman primate models. Nephrons do not regenerate; so the nephrons present at birth must last a lifetime. Prematurity causes reduction of nephron endowment with lowering gestational age.

Survivors of extreme prematurity suffer arrested development of organs with reduced nephron endowment as a consequence of hypoxic-ischemic and nephrotoxic renal insults. Short-term consequences include electrolyte disbalances, acidosis, and impaired free water handling. These could potentially result in prolonged respiratory support, growth failure, and suboptimal neurodevelopmental outcomes in the short term. In later life, subclinical chronic renal insufficiency may progress even in the asymptomatic survivors [90].

Kidney size and nephron number are known to be reduced in surviving premature infants due to disruption of organogenesis at a crucial developmental time point. Progressive kidney disease in individuals born prematurely is multifactorial with involvement of genetic and environmental events that contribute to the programming of subsequent risks of cardiovascular risks and renal disease. Nephrotoxic medication further may decrease renal function of premature neonates.

## 8. Conclusions and Further Perspectives

Excessive or inappropriate activation of complement is a key factor in many diseases [2,8,12,15,16,28,29,30,31,32,36,37,38,39,40,54,55,66,67,70,74,79,84,91,92,93,94]. Classical complementopathies, such as PNH and aHUS have been extensively studied and have served as model diseases for the development of targeted therapy by complement inhibitors.

PNH and aHUS are both diseases caused by mutations with the consequent complement-mediated cell destruction [40,67,92,93,94] and renal involvement. Eculizumab has revolutionized the treatment of these diseases and considerably improved the overall outcome and quality of life with beneficial effect on the renal function. Additionally, pregnancy of women with such diseases have been discouraged, but the eculizumab for both prevention of disease exacerbation and treatment of disease-related complications gave hope for many with the encouraging results on the preservation of the renal function [78,79].

There is considerable evidence of the involvement of inappropriate complement activation in certain other diseases that may be life threatening (APS, SCD, HELLP) for both mother and fetus/neonate. Although scarce, the encouraging results on the off-label use of eculizumab in such diseases gives a further hope that this complement inhibitor will become a part of treatment protocol. This would enable safe [17,71,84,95] pregnancy prolongation, lower perinatal mortality, better life quality and decreased rate of prematurity–related acute and chronic renal failure.

However, not only the very high price of eculizumab, lack of standardized treatment protocols (dosage, infusion intervals, length and monitoring of therapy) for particular diseases and consensus in determination of the most suitable complement markers and genetic tests (expensive, time-consuming, and not immediately available to help guide clinical practice at presentation) are just some of the obstacles in the rapid implementation of this drug into the clinical medicine for off-label use [2,39].

Apart from the lack of large studies on the use of eculizumab for off label indications, one of the most important stumbling blocks for the wider use of this drug is its extremely high price. It has been estimated that the annual treatment cost of eculizumab for aHUS is $700,000. However, despite the expected cost-effectiveness of eculizumab treatment after kidney transplantation in patients with aHUS demonstrated by van den Brand et al. [96], total costs for any eculizumab-based treatment regimen remain high. Authors speculate that price reductions for eculizumab may need to be negotiated in order to increase its acceptability. None of the markers for aHUS recurrence or treatment response have been validated in clinical studies. Defining and validation of such markers for aHUS recurrence and eculizumab treatment response would help fine-tune therapies and will probably result in improved cost-effectiveness of eculizumab-based treatment strategies [96].

Similar cost-effectiveness analysis for PNH reported that eculizumab may provide substantive benefits to patients with PNH in terms of life expectancy and quality of life but at a high incremental cost and a substantial opportunity cost [97].

The nature of rare diseases makes the application of cost-effectiveness analysis difficult. Furthermore, the evidence on the efficacy of new interventions is usually limited, which, combined with a lack of long-term natural history data relating to rare diseases and their heterogeneous nature, makes estimating long-term survival and quality of life problematic.

Of note, possible serious side-effects associated with eculizumab treatment may be life-threatening, limit its use or cause discontinuation of the therapy. The most common side-effect is a headache that is experienced in more than one in ten patients receiving eculizumab. Serious complications (meningococcal infections, sepsis, peritonitis) are uncommon (≥1/1000 to <1/100) but life-threatening. Prior to initiating eculizumab therapy, it is recommended that PNH, and aHUS patients initiate immunizations according to current immunization guidelines. Additionally, all patients must be vaccinated against meningococcal infections at least 2 weeks prior to receiving eculizumab unless the risk of delaying eculizumab therapy outweighs the risks of developing a meningococcal infection. Patients who initiate eculizumab treatment less than 2 weeks after receiving a meningococcal vaccine must receive treatment with appropriate prophylactic antibiotics until 2 weeks after vaccination [98,99]. However, infectious morbidity of pregnant women treated with eculizumab is not alarming. Data on 75 pregnancies in 61 women with eculizumab-treated PNH reported two cases of postpartum sepsis and no cases of serious neonatal infectious morbidity [71]. The latter may be partially explained by the fact that eculizumab treatment during pregnancy does not affect the complement system activity of the newborn [72]. It is plausible to conclude that meningococcal vaccine is recommended also for all patients on eculizumab for off-label indications. Potential medico-legal issues should not be neglected either.

**Figure 2:**
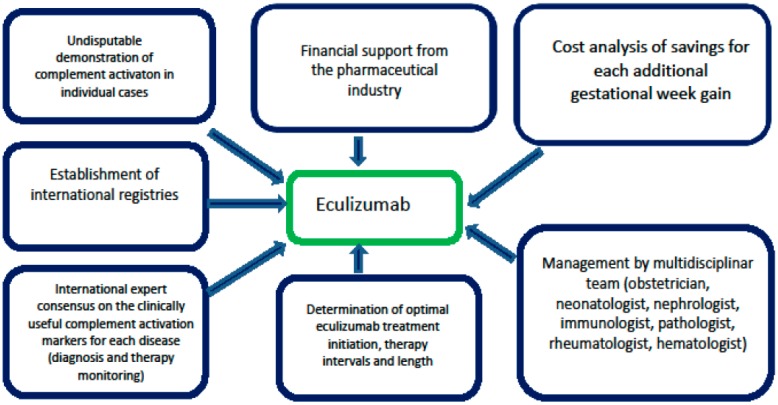
Essential steps before potential extended use of eculizumab in complement-associated diseases in pregnancy.

Concerning rarity of such diseases, especially during pregnancy, one of the steps to gain more knowledge would be International registries of case-reports and well-designed multicenter trials. Unless the pharmaceutical industry recognizes the potential gold mine, particularly in preeclampsia /HELLP (with the incomparably higher incidence than the other diseases discussed in this review) and provide a financial support for the further research and hopefully authority approval, it may be possible that the high price will remain the main obstacle for future progress (Figure 2). Similar concerns appear also for potential extended use of eculizumab for off-label indications in non-pregnant patients, as well.

More research is urgently needed to develop new treatments and preventive modalities, particularly in pregnancies complicated by maternal preexisting comorbidities and risk factors for kidney injury that show increasing prevalence in the developed countries. Development of precise complement functional and genetic studies with rapid turnaround time is urgently needed [39,95,100,101].

By potentially preventing extremely preterm birth, one of the most devastating pregnancy complications, eculizumab might also in this way decrease the risk of chronic kidney disease and cardiovascular diseases of the offspring. Multidisciplinary teams should be involved not only in pregnancy planning of women with preexisting chronic diseases associated with complement activation, but also in treatment of complications in such pregnancies [39,95,100,101].

Of note, several novel drugs have been recently developed for blocking the complement cascade, including purified plasma proteins (purified factor H, C1 inhibitor) new monoclonal antibodies (Anti-C1s, Anti-factor D), recombinant proteins (complement receptor 1 and targeted complement regulatory proteins), small molecules (e.g., compstatin), and small interfering RNA agents [102]. We hope that the use of eculizumab for extended indications during pregnancy will be a reality in the near future.

## Figures and Tables

**Table 1:** Key references demonstrating complement activation in antiphospholipid syndrome, sickle-cell disease, and HELLP syndrome (potential extended indications for eculizumab use).

Disease	Subject(s)	Treatment	Key Points	Ref.
Antiphospholipid syndrome	Pregnant mice	Human IgG containing aPL antibodies	Antibodies or peptides that block C5a-C5a receptor interactions prevent pregnancy complications.	[8]
	Non-pregnant mice	IgG-APS/rEV576 (treatment group) and IgG-APS/phosphate buffer (controls)	Mice treated with IgG-APS/rEV576 [(coversin), a recombinant protein inhibitor of complement factor 5 activation] had significantly smaller thrombi than those treated with IgG-APS/phosphate buffer. This confirmed involvement of complement activation in antiphospholipid antibody-mediated thrombogenesis and suggested that this effect might be ameliorated by complement inhibition.	[9]
	Pregnant woman (case report)	Eculizumab	Triple positive aPL. Multiple previous arterial thromboses and ongoing ischemia during pregnancy. Significant risk of catastrophic APS. Eculizumab was administered twice before cesarean section which was performed at 32^+4^ gestational weeks with the birth of a heathy child. Complement activity surprisingly increased to normal levels within a week after both doses of eculizumab despite the evidence of complete inhibition of complement activity after each infusion. Pregnancy may influence pharmacodynamics and pharmacokinetics of eculizumab. Individual approach suggested.	[10]
	Pregnant woman (case report)	Eculizumab	Triple positive aPL. Abruptly developed microangiopathic hemolytic anemia, renal insufficiency, and thrombocytopenia at 30^+6^ weeks despite treatment with ASA, LMWH and hydroxycholoquine. Eculizumab was administered and pregnancy was safely continued for 9 days. The second eculizumab infusion was administered a week after the first treatment with rapid normalization of platelet count, renal function and hemoglobin level.	[11]
Sickle-cell disease	Patients with thrombotic microangiopathies	Eculizumab	Increased complement activation was demonstrated in a portion of patients, especially older patients and those with higher HbS levels. In vitro study on the efficacy of complement inhibition by eculizumab in the modified Ham test. Mixing eculizumab-containing serum with complement-activated sera abolished complement-mediated cell killing in a dose-dependent relationship that was consistent across the three patients tested.	[12]
	SCD patient who developed aHUS (case report)	Eculizumab	Respiratory and renal insufficiency unresponsible to plasmapheresis successfully treated with eculizumab. A clear evidence of complement activation was demonstrated by increased levels of sC5b-9 levels retrospectively analyzed from stored plasma prior to plasmapheresis. After eculizumab treatment and improvement of clinical symptoms and laboratory baseline, sC5b-9 levels normalized. This case demonstrated that patients with SCD may develop complement-mediated thrombotic microangiopathy i.e., aHUS, particularly when an underlying genetic defect in complement regulation is present.	[13]
	Pregnant woman (case report)	Eculizumab	A pregnant woman with HbSS who presented with hyperhemolysis at 25 gestational weeks and worsening anemia despite methylprednisolone and immunoglobulin treatment. Eculizumab treatment resulted in resolution of the hemolysis, with safe delivery at 34 weeks of gestation.	[14]
HELLP syndrome	Sixteen pregnant women with HELLP	N/A	Incresed complement activation demonstration in sera.	[15]
	Pregnant women with HELLP	N/A	Increased complement activation was observed in participants with classic or atypical HELLP compared with those with normal pregnancies and nonpregnant controls. Mixing HELLP serum with eculizumab-containing serum resulted in a significant decrease in cell killing compared with HELLP serum alone. Data of this study demonstrated striking similarities between HELLP and aHUS.	[16]
	Pregnant women with HELLP (case report)	Eculizumab	Prolongation of pregnancy from 26^+3^ to 28^+6^ (17 days) was achieved. In this case, reduction of soluble C5b-9 in plasma and urine correlated with clinical improvement, and resolution of hemolysis, thrombocytopenia and liver inflammation along with a prolonged pregnancy.	[17]
